# An explanation of how mutant and wild-type mitochondria might stably co-exist in inherited mitochondrial diseases

**DOI:** 10.1093/pnasnexus/pgac192

**Published:** 2022-09-16

**Authors:** Axel Kowald, Felix P Kemeth, Tom B L Kirkwood

**Affiliations:** UK National Innovation Centre for Ageing, The Catalyst, 3 Science Square, Newcastle University, Newcastle upon Tyne NE4 5TG, UK; Rostock University Medical Center, Institute for Biostatistics and Informatics in Medicine and Aging Research (IBIMA), 18057 Rostock, Germany; Physik-Department, Nonequilibrium Chemical Physics, Technische Universität München, James-Franck-Str. 1, D-85748 Garching, Germany; UK National Innovation Centre for Ageing, The Catalyst, 3 Science Square, Newcastle University, Newcastle upon Tyne NE4 5TG, UK; Center for Healthy Aging, Department of Cellular and Molecular Medicine, University of Copenhagen, 2200 Copenhagen N, Denmark

**Keywords:** mitochondrial disease, mathematical model, aging

## Abstract

Mitochondria are cellular organelles of crucial relevance for the survival of metazoan organisms. Damage to the mitochondrial DNA can give rise to a variety of mitochondrial diseases and is thought also to be involved in the aging process. The fate of mtDNA mutants is controlled by their synthesis as well as degradation and mathematical models can help to better understand this complex interplay. We present here a model that combines a replicative advantage for mtDNA mutants with selective degradation enabled by mitochondrial fission and fusion processes. The model not only shows that the cell has efficient means to deal with (many) types of mutants but, surprisingly, also predicts that under certain conditions a stable co-existence of mutant and wild-type mtDNAs is possible. We discuss how this new finding might explain how mitochondria can be at the heart of processes with such different phenotypes as mitochondrial diseases and aging.

Significance Statement Mitochondria are involved in both mitochondrial diseases and the aging process. However, while mitochondrial mutants accumulate progressively inside affected cells during aging, in mitochondrial diseases mutants and wild-type seem to co-exist for several decades. We present here a mathematical model that describes the mitochondrial replication process as well as selective degradation enabled by mitochondrial fission and fusion processes. Interestingly, the model shows that under certain conditions this enables a stable co-existence of mutant and wild-type. We believe that this finding might explain how mitochondria can cause the different phenotypes seen in mitochondrial diseases and aging.

## Introduction

The replication dynamics of mitochondria are intriguing. During the long co-evolutionary history of this organellar symbiont, it is thought that significant parts of the primordial mitochondrial genome (mtDNA) were transferred to the nucleus of the host cell, which helps to ensure suitable control of the intracellular mitochondrial population (1). Nevertheless, mitochondria remain as distinct genetic entities with a life cycle of birth (formation) and death (destruction) of their own. This means that they are subject to Darwinian evolution not only across long evolutionary timescales as components of eukaryotic organisms, but also within the shorter timescale that is relevant to their “ecological niche” as defined by the spatial (body) and temporal (lifespan) bounds of the individual organism.

The mutation rate of mtDNA is high, relative to that of nuclear DNA (2), and thus variation is a regular feature of mitochondrial populations within individual cells. The term “heteroplasmy” denotes the co-existence of multiple mtDNA variants within a single cell. Furthermore, there is strong evidence that the burden of mtDNA mutations increases across the life course (3, 4), which has resulted in much interest in the possibility that mtDNA mutations play a causative role in the aging process. In this scenario, the initial burden of mtDNA mutations in the new generation is believed to be kept low through quality control processes operating within the germ cell (oocyte) lineage, but increases somatically, thereafter. Specifically, in tissue sections of old individuals a mosaic pattern of healthy and affected cells can be observed. In the affected cells, mtDNA deletion mutants have accumulated and represent a substantial fraction of the mitochondrial population. This phenomenon has been found in different mammalian species like mice, rats, monkeys, and humans (5–12). Interestingly, inside individual cells, the mitochondrial population is frequently overtaken by a single type of mutant. This forms the basis for the mitochondrial theory of aging (13–15).

However, mtDNA mutations are also known to be associated with certain human inherited diseases, where pre-existing mtDNA mutations in the mother are transmitted via the germ line to her affected progeny. Mitochondrial diseases can arise through mutations of the mitochondrial genome (called primary mtDNA diseases) or via mutations of nuclear genes affecting mitochondrial functions. Primary mitochondrial diseases can be caused by point mutations or deletions and among the most common of such diseases are Leber’s hereditary optic neuropathy (LHON), mitochondrial encephalomyopathy, lactic acidosis, and stroke-like episodes (MELAS), myoclonus epilepsy with ragged-red fibres (MERRF), Kearns–Sayre syndrome (KSS), and chronic progressive external ophthalmoplegia (PEO) (16–18). In such cases, the individual burden in terms of copy number of mtDNA mutants can remain reasonably stable for many years, although there is often a tendency for pathological effects to worsen with age.

Within the framework of Darwinian evolution, two main factors are responsible for changes in gene frequencies. One is selection, which can act through either the birth rate (formation of new copies) or death rate (removal of existing copies). The second is drift, whereby stochastic fluctuations can lead to alteration in gene frequencies through the actions of chance. By its nature, the existence of drift is hard to demonstrate experimentally. Drift must exist, at least theoretically, and mathematical modelling has shown that it could be sufficient to explain the observed somatic expansion of mutant mtDNAs in the case of long-lived organisms such as humans (19, 20). It is unable, however, to explain comparable data in short-lived species such as mice (21, 22). We will therefore focus our present attention on selection.

Regarding selection, multiple suggestions have been made to account for the age-related increase in somatic mtDNA mutations by selective means. These have been motivated particularly by the observation that within individual cells the mitochondrial population is, as mentioned, commonly overtaken by a single mutant type, very often a deletion in which a part of the normal mtDNA genome has been lost. Recent evidence also indicates that a continual cycle of mitochondrial fusion–fission events, whereby single mitochondria combine into a fused syncytium only to break apart again by fission, provides important scope for selection to occur. Within the fusion–fission cycle, selection can occur during fusion through factors that increase or decrease the likelihood that mutant mitochondria are included. There is also good evidence that selection occurs after fission through different likelihoods of wild-type and mutant mitochondria being degraded via mitophagy (23–27).

Given the support for the idea that mutations of the mitochondrial DNA are involved in such different processes as mitochondrial diseases and aging, it is interesting to ask how, in the context of mitochondrial diseases, is it possible to explain that a stable heteroplasmic co-existence of wild-type and mutant mitochondria appears to exist, while, in the case of aging, mutant levels continuously rise inside cells after the initial mutation event. To address this question, we extend here our model that explains how the mitochondrial transcription system could confer a selection advantage to mitochondrial deletion mutants (22, 28) by including reactions that describe the fission and fusion process.

There have been a few models that combined mitochondrial replication and degradation with fission and fusion (29–32), but all those models concentrate on a replication process without selection advantage for mutants. Furthermore, the number of mtDNAs is assumed to be absolutely fixed (29), replication rate is a constant (30, 31) or replication rate is adjusted in such a way that the system is steered towards a particular copy number (32). Our idea about replication control is quite different in that we propose that the deletion of genes involved in a transcriptional feedback loop also leads to a replication advantage of such mtDNAs. We also model mitochondrial copy number in more biological terms by assuming that the cell tries to maintain a certain ATP level and not a certain copy number of mitochondria. This also agrees nicely with experimental findings that in muscle fibres, which are taken over by mitochondrial mutants the total number of mtDNAs is greatly increased and not fixed (9). Surprisingly, the new model identifies conditions under which a stable co-existence of mutant and wild-type mtDNAs can be expected, which no model has predicted previously. We discuss the relevance of this finding for the role of mutant mitochondria in ageing as well as in diseases.

## Model development

Fission and fusion leads to a complex mitochondrial network that can be modeled in a spatial way and studied by computer simulations (30, 31) or it can be described in a way ignoring spatial information by categorizing mtDNAs as either being in a fused or unfused state (called “singleton”) (32). We follow here the latter approach, which is more amenable to analytic analysis. We therefore extended our current model (22) by the reactions described in Aryaman et al. (32), resulting in a model with five variables, namely wild-type in the fused (wtf) and singleton (wts) states, mutants in the fused (mtf) and singleton (mts) states as well as ATP.

**Fig. 1.**
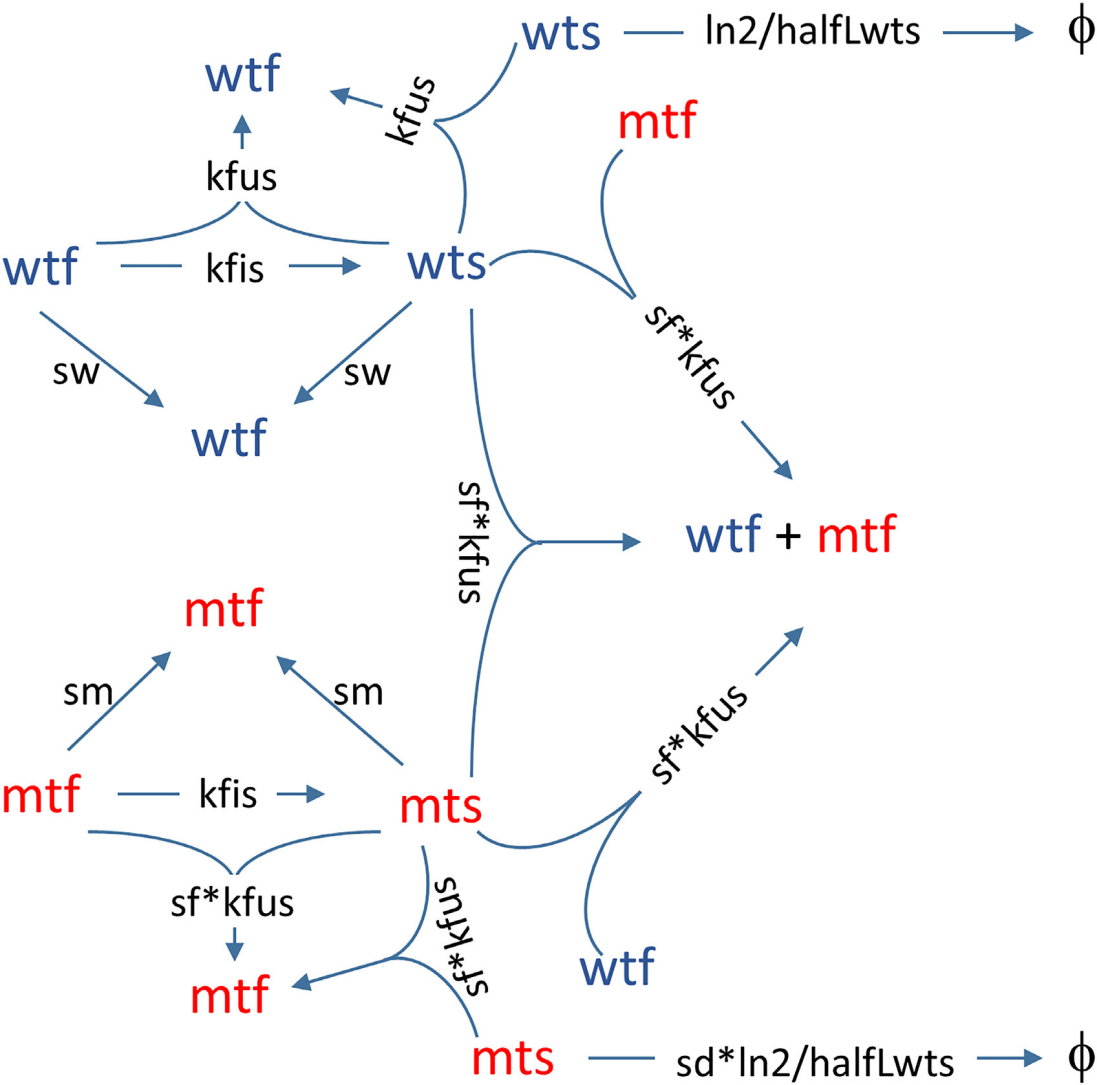
Schematic reaction network of mtDNAs that is described by our model. It encompasses fission and fusion processes of wild-type in the fused (wtf) and singleton (wts) states, as well as mutant mtDNA in the fused (mtf) and singleton (mts) states. It also contains degradation and synthesis reactions and important parameters involved in the various reactions. For details on parameters, see Table 1 and the main text for a detailed description of the reactions. Please note that ATP has been omitted from the diagram for the sake of clarity.

Fig. 1 shows the resulting reaction network of the four different types of mtDNAs. Please note that for clarity the same variable can appear at multiple places in the diagram and also that ATP has been omitted. In the upper half reactions of wild-type mtDNAs are shown, which are mirrored in the bottom half for mutant mtDNAs. For instance, a wild-type mtDNA in the fused state (wtf) can undergo fission (depending on parameter *kfis*) and enter the singleton state (wts). Conversely, *wts* can react with *wts* or *wtf* and fuse into two *wtf*, depending on the parameter *kfus* (stoichiometry is ignored in the diagram). The fused and singleton forms of mtDNA can undergo replication (depending on rate parameters *sw* and *sm*), but in both cases this will result in mtDNA molecules in the fused state [as in Aryaman et al. (32)]. Degradation is assumed to occur via mitophagy, which can only act on small fragments of the mitochondrial network, i.e. singletons. Thus, only *wts* and *mts* undergo degradation reactions in our model.

Wild-type and mutant can in principle perform an identical set of reactions but the parameter values influencing some of these reactions are different. There are two principal mechanisms a cell can employ to suppress mutant mtDNAs. Defective mitochondria can selectively be degraded and they can preferentially be kept in the singleton state (where degradation takes place). Both strategies are being used and the molecular details are now fairly well understood (25–27). Normal mitophagy relies on the PINK and Parkin system. Without membrane potential, the kinase PINK accumulates on the outer mitochondrial membrane (OMM) and phosphorylates ubiquitin (that is connected to OMM proteins). Parkin is then attracted to those ubiquitins and becomes then phosphorylated and thus activated by PINK. Parkin then ubiquitinates various proteins including mitofusins 1 and 2 (Mfn1/2), which are important for mitochondrial fusion. This leads to degradation of Mfn1/2, prevents fusion, and triggers mitophagy of the affected organelles since the autophagosome binds to the phosphorylated ubiquitins.

We model this using a parameter called “*specificity of degradation*” (*sd* > 1) that increases the mitophagy rate of *mts* and a parameter called “*specificity of fusion*” (*sf* < 1) that lowers the fusion rate of mutant mtDNAs. These mechanisms counteract the higher replication rate of mutant mtDNA, which is expressed in our model by having *sm* > *sw*. Table 1 provides a list of all parameters used together with their standard value and a short description of their meaning.

**Table 1.** Parameters and standard values used for the simulations.

**Name**	**Value**	**Description**
*halfLwts*	10 d	Half-life of wild-type mtDNA in the singleton state. Based on data by Gross et al. (35), Huemer et al. (36), and Korr et al. (37), we chose a half-life of 10 d as standard value.
*sw*	24 d^−1^	Parameter controlling the synthesis rate of wild-type mtDNA. For ATP = 0, this results in a synthesis rate of one mtDNA per hour, based on findings of Clayton (38) and Berk and Clayton (39).
*sm*	36 d^−1^	Parameter controlling the synthesis rate of mutant mtDNA. For ATP = 0, this results in a synthesis rate of 1.5 mtDNA per hour. This assumption is based on our earlier work model (22).
*c*	0.648	Carry capacity of ATP. Calculated such that without mutants the steady-state of wild-type mtDNA is equal to 1,000.
*f*	2.4 d^−1^	Parameter describing how much ATP is produced by wild-type mtDNAs (free parameter).
*v*1	4.8 d^−1^	Parameter controlling how much ATP is consumed by cellular processes (free parameter).
*v*2	0.24 d^−1^	Parameter controlling how much ATP is consumed as maintenance cost per existing mtDNA (free parameter).
*kfis*	10 d^−1^	Fission rate of fused mitochondria [comparable to the value used by Aryaman et al. (32)].
*kfus*	0.01 d^−1^	Fusion rate of mitochondria [comparable to the value used by Aryaman et al. (32)].
*sf*	0.5	Parameter (“specificity of fusion”) specifying by how much the fusion rate of mutant mitochondria is reduced compared to wild-type (free parameter).
*sd*	1.1	Parameter (“specificity of degradation”) specifying by how much the degradation rate of singleton mutant mtDNAs (mts) is increased over wild-type (free parameter).

With the reaction scheme (Fig. 1) and the table of parameters, it is now possible to develop a set of ordinary differential equations (ODE) that describe the time course of the five model variables (*wtf, wts, mtf, mts*, and ATP). The first terms of Eqs. 1 and 3 describe the synthesis of wild-type and mutant mtDNAs, whereas the last terms of Eqs. 2 and 4 describe their degradation (which happens only in the singleton state). Synthesis is proportional to the synthesis parameters *sw* and *sm*, and negatively regulated by the ATP level. If the ATP level is equal to the constant “*c*,” the synthesis rate drops to 50% of its maximal value. Degradation is controlled by the parameter *halfLwts* and somewhat larger for mutants via the parameter “*sd*.” The other terms in Eqs. 1–4 describe the effects of various fission and fusion reactions that redistribute mtDNAs between the fused and singleton states (see also Fig. 1).

The equation for ATP (Eq. 5) comprises three terms, the first describing the production by wild-type mtDNA and the other two describing its consumption. The last term reflects our assumption that the mere existence of mtDNA molecules imposes some energetic costs: because mRNA and proteins are synthesized from it, new membrane has to be synthesized to accommodate the proteins, and all these components also have to be degraded again. ATP is also consumed by many other processes in the cell, which is represented as an aggregate by the second term.

(1) $$\begin{eqnarray*}{\rm{\ }}\frac{{dwtf}}{{dt}} &=& \frac{{sw \cdot \left( {wtf\left( t \right) + wts\left( t \right)} \right)}}{{\frac{{ATP\left( t \right)}}{c} + 1}}{\rm{\ }} + kfus \cdot wts{\left( t \right)}^2 + kfus \cdot wts\left( t \right) \cdot wtf\left( t \right)\nonumber\\&& + sf \cdot kfus \cdot wts\left( t \right) \cdot \left( {mtf\left( t \right) + mts\left( t \right)} \right) - kfis \cdot wtf\left( t \right),\end{eqnarray*}$$

(2) $$\begin{eqnarray*}{\rm{\ }}\frac{{dwts}}{{dt}} &=& {\rm{\ }} - kfus \cdot wts{\left( t \right)}^2 - kfus \cdot wts\left( t \right) \cdot wtf\left( t \right)\nonumber\\&& - sf \cdot kfus \cdot wts\left( t \right) \cdot \left( {mtf\left( t \right) + mts\left( t \right)} \right)\nonumber\\&& + kfis \cdot wtf\left( t \right) - wts\left( t \right) \cdot {\rm{ln}}2/{\rm{\mathit{ halfLwts}}},\end{eqnarray*}$$

(3) $$\begin{eqnarray*}{\rm{\ }}\frac{{dmtf}}{{dt}} &=& \frac{{sm \cdot \left( {mtf\left( t \right) + mts\left( t \right)} \right)}}{{\frac{{ATP\left( t \right)}}{c} + 1}}{\rm{\ }} + sf \cdot kfus \cdot mts{\left( t \right)}^2 + sf \cdot kfus \cdot mts\left( t \right)\nonumber\\&& \cdot mtf\left( t \right) + sf \cdot kfus \cdot mts\left( t \right) \cdot \left( {wtf\left( t \right) + wts\left( t \right)} \right)\nonumber\\&& - kfis \cdot mtf\left( t \right),\end{eqnarray*}$$

(4) $$\begin{eqnarray*}{\rm{\ }}\frac{{dmts}}{{dt}} &=& {\rm{\ }} - sf \cdot kfus \cdot mts{\left( t \right)}^2 - sf \cdot kfus \cdot mts\left( t \right) \cdot mtf\left( t \right)\nonumber\\&& - sf \cdot kfus \cdot mts\left( t \right) \cdot \left( {wtf\left( t \right) + wts\left( t \right)} \right)\nonumber\\ && + kfis \cdot mtf\left( t \right) - mts\left( t \right) \cdot sd \cdot {\rm{ln}}2/{\rm{\mathit{ halfLwts}}},\end{eqnarray*}$$

(5) $$\begin{eqnarray*}{\rm{\ }}\frac{{dATP}}{{dt}} &=& {\rm{\ }}f \cdot \left( {wtf\left( t \right) + wts\left( t \right)} \right) - v1 \cdot ATP\left( t \right)\nonumber\\ && - v2 \cdot \left( {wtf\left( t \right)+ wts\left( t \right) + mtf\left( t \right) + mts\left( t \right)} \right).\end{eqnarray*}$$

## Steady-state analysis without synthesis and degradation

To get some first insights into the model behavior, it is useful to calculate the steady-state levels while ignoring for the moment synthesis and degradation of mitochondria. This is a reasonable approximation since mitochondrial dynamics happens at a much faster time scale (minutes) compared to synthesis and degradation (days).

Under that condition, ATP no longer influences Eqs. 1–4, which can be rearranged to yield the following steady-state expressions with *wt* = *wtf* + *wts* and *mt* = *mtf* + *mts*:

(6) $$\begin{equation*}
wtf\ = {\rm{\ }}\frac{{kfus \cdot wt \cdot \left( {sf \cdot mt + wt} \right)}}{{kfis + kfus \cdot \left( {sf \cdot mt + wt} \right)}},
\end{equation*}
$$

(7) $$\begin{equation*}
wts\ = {\rm{\ }}\frac{{kfis \cdot wt}}{{kfis + kfus \cdot \left( {sf \cdot mt + wt} \right)}},
\end{equation*}
$$

(8) $$\begin{equation*}
mtf\ = {\rm{\ }}\frac{{kfus \cdot mt \cdot sf \cdot \left( {mt + wt} \right)}}{{kfis + kfus \cdot sf \cdot \left( {mt + wt} \right)}},
\end{equation*}
$$

(9) $$\begin{equation*}
mts\ = {\rm{\ }}\frac{{kfis \cdot mt}}{{kfis + kfus \cdot sf \cdot \left( {mt + wt} \right)}}.
\end{equation*}
$$

From this, the fraction of mutant and wild-type mtDNA that are in the single state can be calculated. Interestingly, the fractions not only depend on constant model parameters, but also on the total number of mutant and wild-type mitochondria that currently exist in the cell. As a consequence, the fraction of mtDNAs in the singleton state drops as the total number of mtDNAs increases. This has important consequences (for the full model) since mitochondria in the fused state are protected from degradation.

(10) $$\begin{equation*}
wtsFrac\ = \frac{{wts}}{{wtf + wts}}{\rm{\ }} = {\rm{\ }}\frac{{kfis}}{{kfis + kfus \cdot \left( {sf \cdot mt + wt} \right)}},
\end{equation*}
$$

(11) $$\begin{equation*}
mtsFrac\ = {\rm{\ \ }}\frac{{mts}}{{mtf + mts}} = \frac{{kfis}}{{kfis + kfus \cdot sf \cdot \left( {mt + wt} \right)}}.\
\end{equation*}
$$

Although we ignore here degradation and synthesis, we can nevertheless say something about the half-lives in the full system. Since degradation only affects the fraction of mitochondria in the singleton state, the half-life averaged over all wild-type mtDNAs is given by *halfLwts/wtsFrac* and the half-life averaged over all mutant mtDNAs is given by *halfLwts*/(mtsFrac*sd), where *sd* is the above mentioned parameter accounting for the preferred degradation of mutant mitochondria. The ratio of those half-lives (*wtmtHalfL*), represents the selection advantage of wild-type over mutant and if it is larger than the replication advantage of mutants (given by the ratio of *sm/sw*), then those are cleared, otherwise mutants accumulate.

(12) $$\begin{equation*}
wtmtHalfL\ = {\rm{\ }}\frac{{sd \cdot \left( {kfis + kfus \cdot \left( {sf \cdot mt + wt} \right)} \right)}}{{kfis + kfus \cdot sf \cdot \left( {mt + wt} \right)}}.
\end{equation*}
$$

As Eq. 12 shows, this selection advantage not only depends on constants like fission and fusion parameters, but also on the total number of mutants and wild-type, which can of course change dynamically with time. Interestingly, if there is no difference in fusion rates (*sf* = 1), the equation simplifies to *wtmtHalfL* = *sd*, which means that the selection advantage of wild-type is completely independent of the actual levels of mtDNAs and simply given by the difference in degradation rates.

**Fig. 2.**
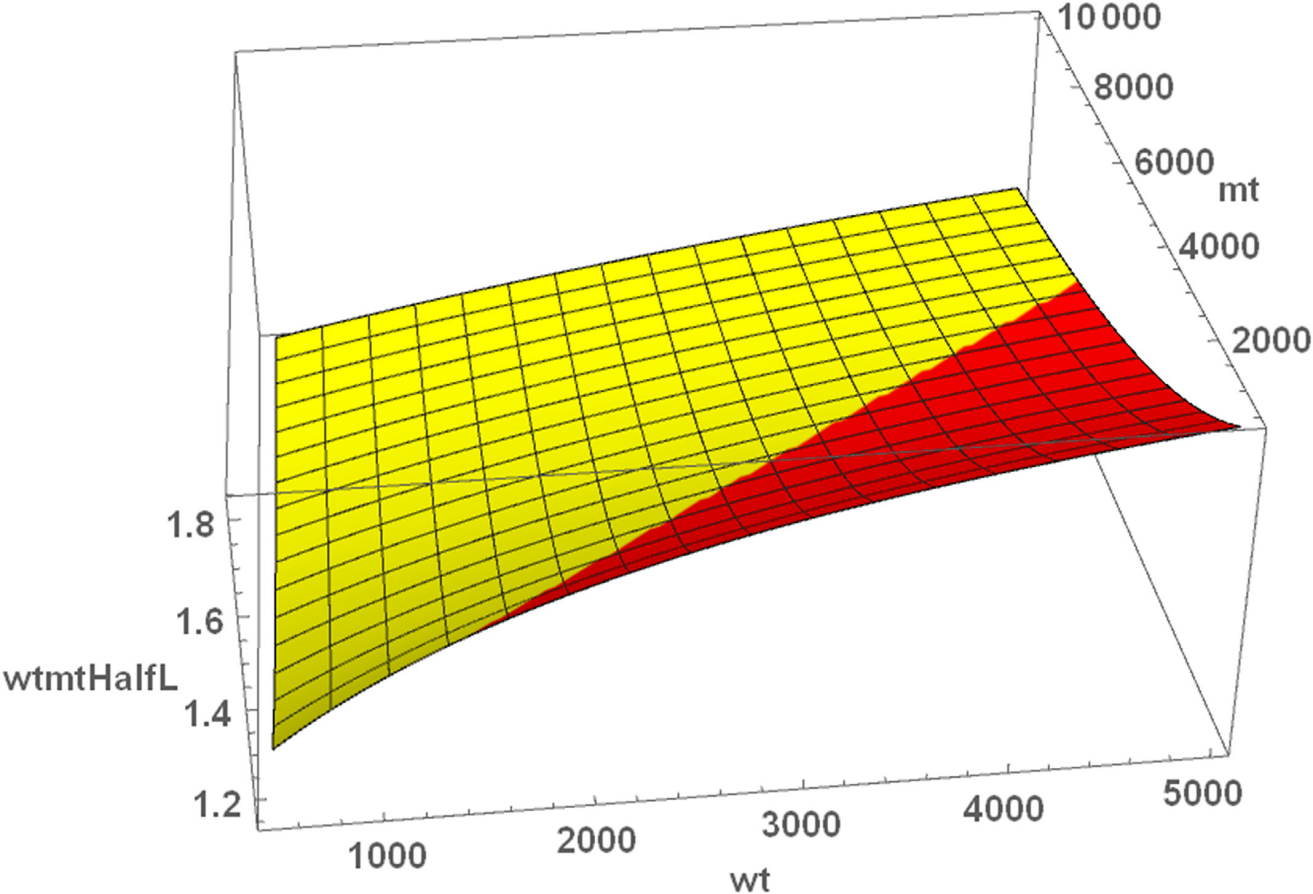
Ratio of *wt* and *mt* half-lives (*wtmtHalfL*) depending on the existing number of wild-type (*wtf* + *wts*) and mutant (*mtf* + *mts*) mtDNAs. In the yellow area, *wtmtHalfL* is below *sm/sw* and mutants can accumulate, in the red area *wtmtHalfL* is above *sm/sw* and mutants are cleared. For the calculation, the standard parameter values of Table 1 were used.

In our earlier work (22), we assumed that mutant mtDNAs have a replicative advantage of 50% (*sm/sw* = 1.5). Eq. 12 is plotted in Fig. 2 with the standard parameter values of Table 1 for a range of wild-type and mutant values. The area where *wtmtHalfL* is below *sm/sw* is shown in yellow and in red where it is above.

## Steady-state solutions of full model

While we ignored synthesis and degradation in the last section, they have to be included to learn about the long term behavior of the system. Surprisingly, the full system (Eqs. 1–5) can be solved analytically for fixed points resulting in four solutions. Apart from the trivial solution (all variables are zero) and a nonphysical solution (negative concentrations), there are also possible solutions for a situation where only wild-type mtDNAs (and ATP) are present as well as for a situation with fix-points for all types of mtDNA. The expressions are rather long and convoluted so that there is not much point in presenting them here. We do, however, list them in the Supplementary Material.

In the following sections, we will explore the stability of these fix-points. If a stable co-existence of wild-type and mutant mtDNAs is possible, this would be of special interest since, to our knowledge, mathematical models of mitochondrial populations so far did not predict this phenomenon and it might explain how defective mitochondria can play important roles in such different processes as aging and mitochondrial diseases.

## Time-course simulations of full model

**Fig. 3.**
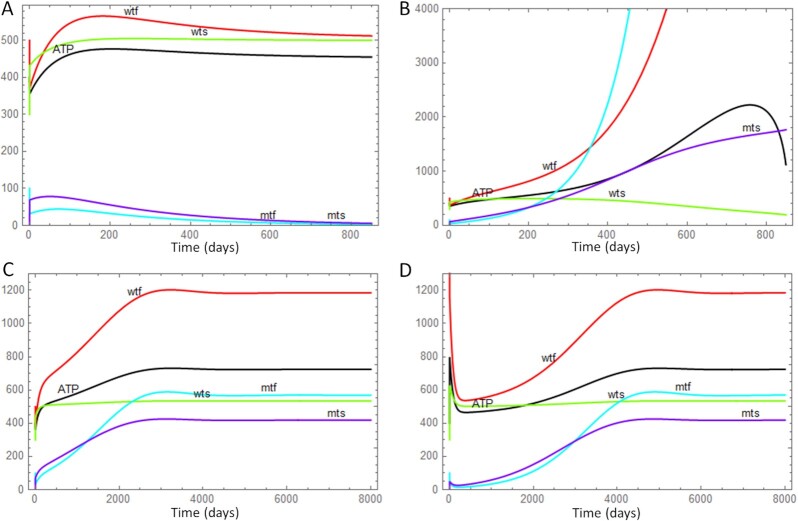
Numerical time-course calculations of the model variables using different parameter values for the specificity of degradation (*sd*). (A) A value of *sd* = 1.2 gives the wild-type a strong selection advantage, leading to the gradual disappearance of mutant mtDNAs (initial values ATP = 400, *wtf* = 500, *wts* = 300, *mtf* = 100, *mts* = 0). (B) With a value of *sd* = 1.0, the selection advantage is reversed and mutants (*mtf* and *mts*) quickly accumulate until the system collapses [initial values as in (A)]. (C and D) An intermediate value of *sd* = 1.1 leads to a system where mutants and wild-type stably co-exist. If the system is started with amounts of wild-type below [initial values as in (A)] (C) or above (initial values ATP = 400, *wtf* = 1500, *wts* = 300, *mtf* = 100, *mts* = 0) (D) the steady state, the system returns to the equilibrium situation. The other parameters are those given in Table 1.

To obtain a first understanding of the dynamical behavior of the model, we performed various time-course simulations of Eqs. 1–5 using the software package Mathematica (Fig. 3). In these simulations, we varied the parameter that controls the specificity of degradation (*sd*), to see which consequences this has for the fate of mutant mtDNAs that are initially present in the system (together with wild-type mtDNAs). Fig. 3A shows that if *sd* is large enough (here 1.2), wild-type mtDNAs have a selection advantage over mutants, with the latter disappearing over time. This long-term behavior corresponds to the steady-state solution that only contains ATP and wild-type mitochondria. If *sd* is reduced to a value of 1.0, the selection advantage disappears and now mutant mtDNAs accumulate caused by their replication advantage given by *sm/sw* (Fig. 3B). This imposes increasing energetic costs and although the system tries to compensate this by an increase of the total number of mtDNA molecules, it leads finally to a decline of the ATP level (black curve). Eventually, the unlimited accumulation of mitochondria causes the system to collapse through ATP exhaustion. However, as predicted by the steady-state analysis, there are also intermediate values of *sd* that allow for the co-existence of mutant and wild-type mtDNAs. This fix point is stable since it is approached from initial values that are below (Fig. 3C) or above (Fig. 3D) the equilibrium amount. The simulations also show some very fast initial spikes. These stem from rapid changes of the ATP level and the network state of mitochondria (fused or singleton). As explained above these reactions happen at a very fast time scale and can hardly be resolved in the long-term simulations of Fig. 3.

**Fig. 4.**
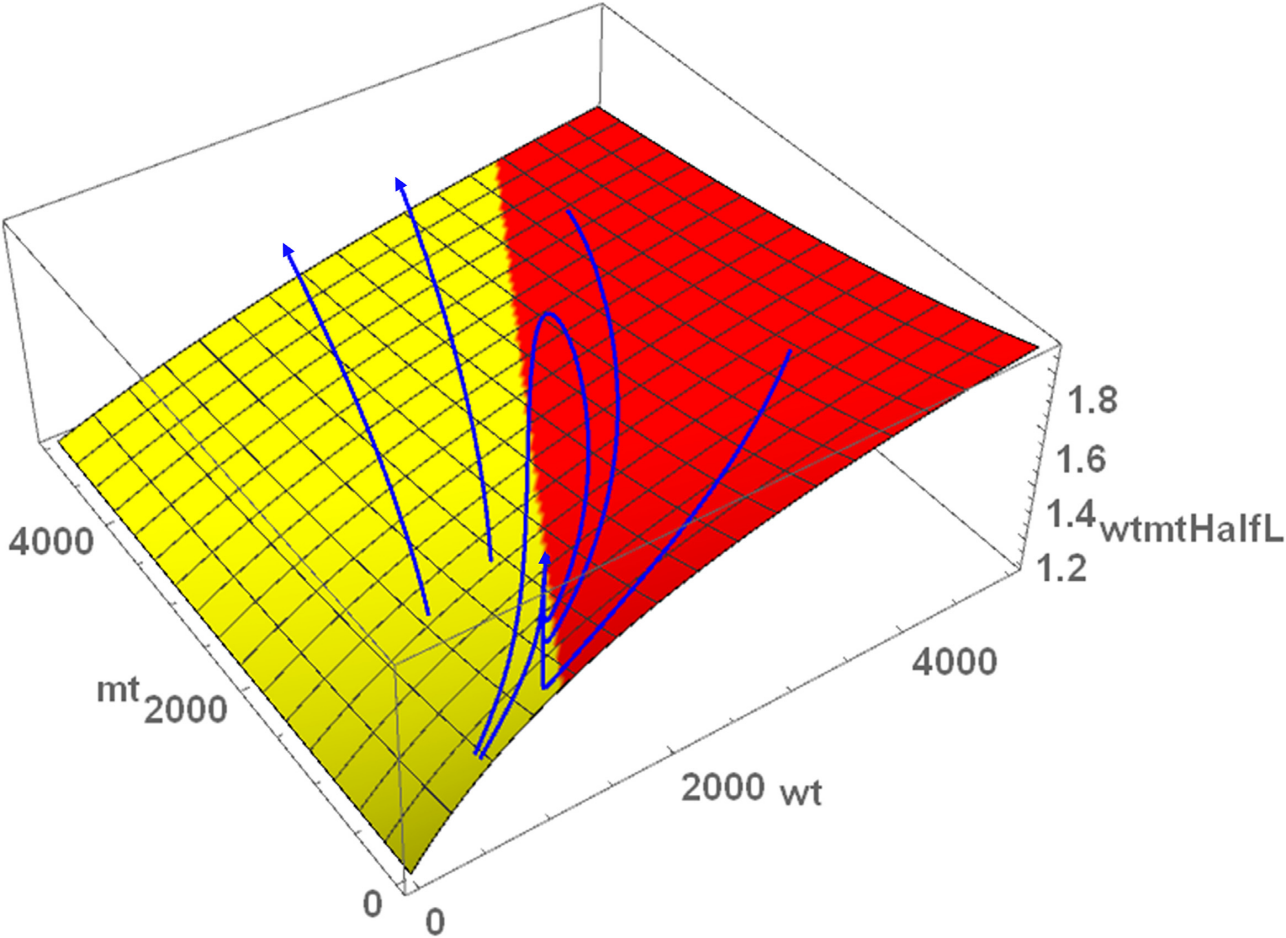
Various time-course trajectories with different starting values (but identical parameter values), superimposed on the function for *wtmtHalfL*. Six time-course simulations were performed (with *sd* = 1.1) with starting conditions shown by the start of the trajectories. Two led to a collapse of the system, while four approached a steady state located at the borderline where *wtmtHalL* = *sm/sw*. The other parameters are those given in Table 1.

When studying the system without synthesis and degradation, it was possible to derive an expression for the ratio of wild-type to mutant half-life (Eq. 12), which represents the selection advantage of wild-type mtDNAs. But that expression alone does not provide insights into the dynamical behavior of the system. We therefore performed various time-course simulations using different starting values and superimposed the trajectories onto a plot of Eq. 12 (Fig. 4). Two of the trajectories led to a collapse of the system, while four converged onto a stable fix point that is located on the boundary where *wtmtHalfL* is equal to the ratio of *sm/sw* (yellow–red borderline). This is possible since the model describes processes that confer a selection advantage to mutants (higher replication rate) as well as processes that confer a selection advantage to wild-type mitochondria [specificity of degradation (*sd*) and specificity of fusion (*sf*)]. Under conditions where both selection advantages are equal and opposite, co-existence is possible. The exact location on the borderline is then further constraint by the condition that also the ATP level has to be at a steady state.

## Reduced model

For a better theoretical analysis of the model behavior, it is helpful to reduce the number of system equations. Luckily, in our case, it is possible to reduce the system to the slow dynamics since the fission and fusion reactions act on a much faster time scale than synthesis and degradation. Thus, for a given number of wild-type and mutant mtDNAs, we use adiabatic approximation to calculate the fraction of fused and singleton mtDNAs. Since the same is true for the ATP level, a reduced model consisting of only two equations for wild-type (*w*) and mutant (*m*) mtDNAs (Eqs. 13–14) can be derived (see the Supplementary Material for details) with the following definitions: $fw\ = \ ( {f - v2} )/( {c \cdot v1} )$, $fm\ = \ - v2/( {c \cdot v1} )$, $\kappa \ = \ kfus/kfis$, $\sigma w\ = \ sw \cdot halfL/ln2$, $\sigma m\ = \ sm \cdot halfL/ln2$, and dimensionless time *t*.

(13) $$\begin{equation*}
\frac{{dw}}{{dt}} = \frac{{\sigma w \cdot w\left( t \right)}}{{1 + fw \cdot w\left( t \right) + fm \cdot m\left( t \right)}}{\rm{\ }} - \frac{{w\left( t \right)}}{{1 + \kappa \cdot \left( {sf \cdot m\left( t \right) + w\left( t \right)} \right)}},
\end{equation*}
$$

(14) $$\begin{equation*}
\frac{{dm}}{{dt}} = \frac{{\sigma m \cdot m\left( t \right)}}{{1 + fw \cdot w\left( t \right) + fm \cdot m\left( t \right)}}{\rm{\ }} - \frac{{sd \cdot m\left( t \right)}}{{1 + \kappa \cdot sf \cdot \left( {m\left( t \right) + w\left( t \right)} \right)}}.
\end{equation*}
$$

**Fig. 5.**
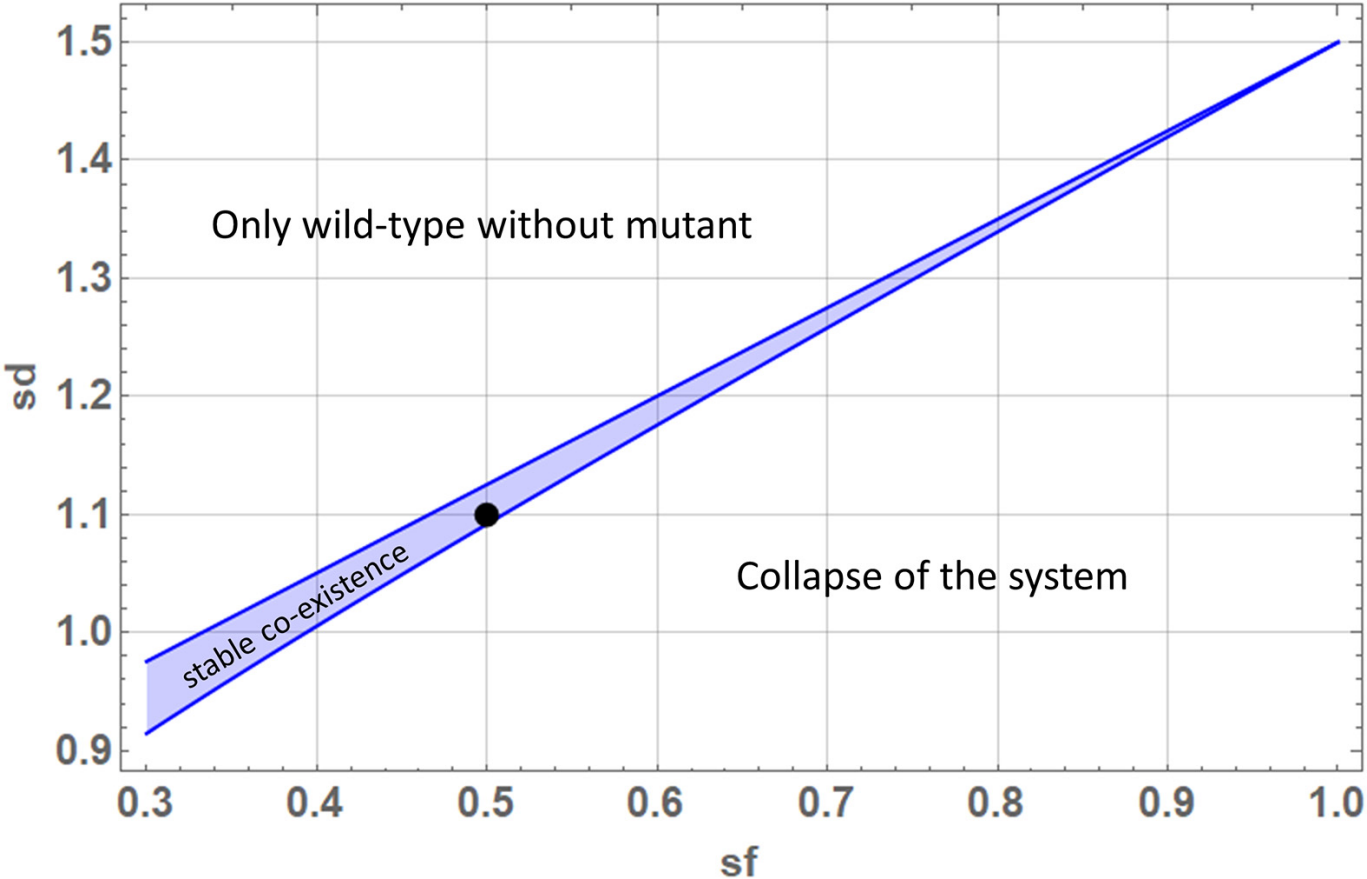
Influence of the specificity of fusion (*sf*) and specificity of degradation (*sd*) on system stability based on analytical expressions derived for the reduced model (see the Supplementary Material). Parameter combinations in the upper left of the diagram lead to a system that only contains *wt* mtDNA, while the blue area indicates the region where mutant and *wt* stably co-exist. The other parameters are those given in Table 1 with the black point representing the standard values for *sd* and *sf*.

Using the reduced system it is possible to find analytic expressions describing the area of stable co-existence of mutant and wild-type mtDNAs, depending on the model parameters (see the Supplementary Material). The *sd* and *sf* are the two principle mechanisms a cell has at its disposal to counteract the accumulation of mutants mtDNAs that possess a replication advantage. Fig. 5 shows that a wide range of values for the parameters *sd* and *sf* leads to a system where the cell successfully fights off mutant mtDNAs (upper left). There is, however, also an area in parameter space, which leads to the collapse of the system and death of the cell (lower right). Those two areas are separated by a smaller region where mutant and wild-type mtDNA stably co-exist. The diagram demonstrates that *sf* and *sd* both are efficient means to combat mutants, and as discussed earlier both approaches are used by the cell (25–27). Interestingly, the region of co-existence becomes smaller and smaller with increasing specificity of fusion and once mutant mtDNAs fuse as efficiently as wild-type (*sf* = 1), co-existence is not possible at all.

**Fig. 6.**
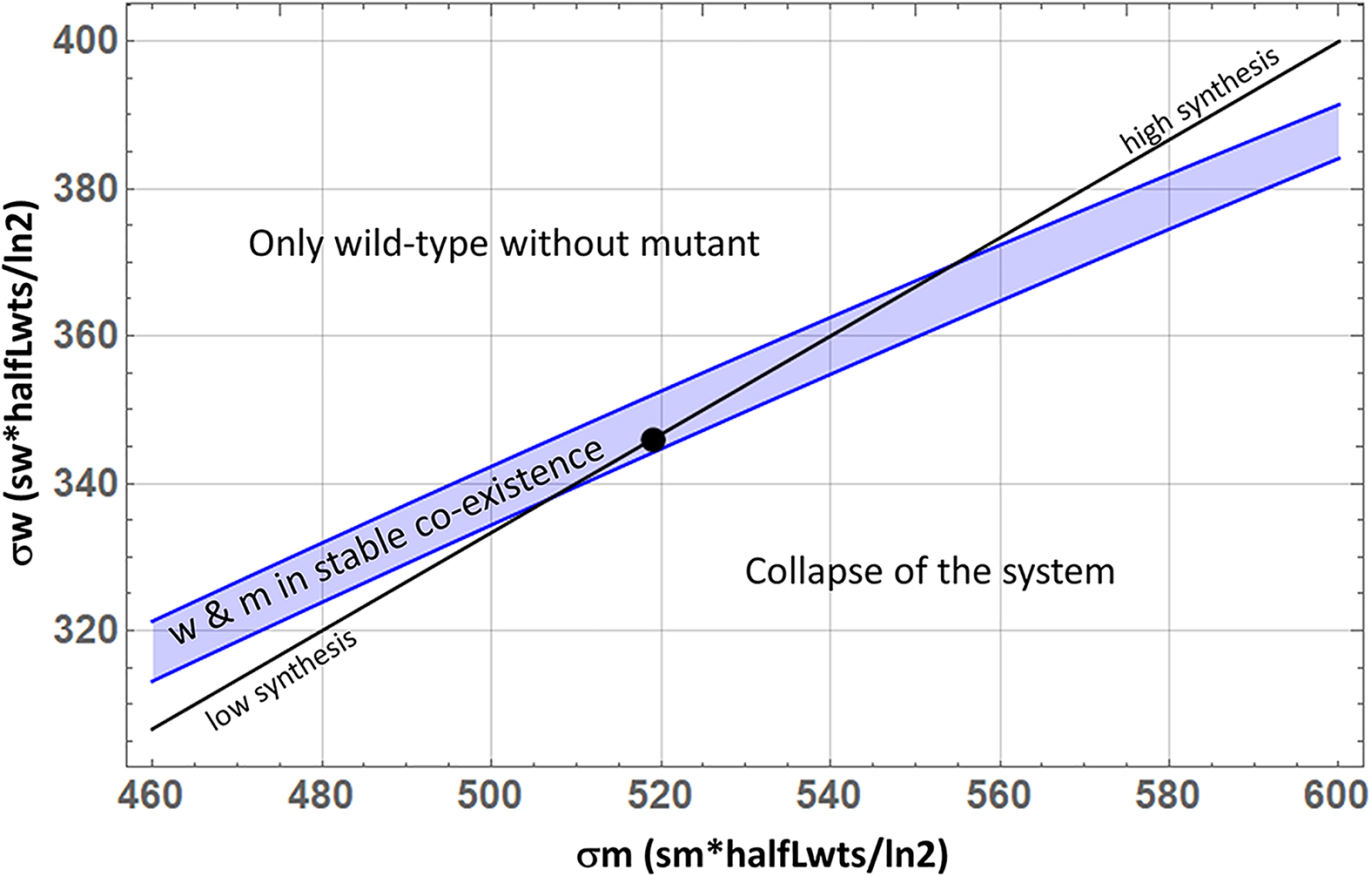
Influence of rescaled growth parameters *σm* and *σw* on system stability based on analytical expressions derived for the reduced model (see the Supplementary Material). Parameter combinations in the upper left of the diagram lead to a system that only contains *wt* mtDNA, while the blue area indicates the region where mutant and *wt* stably co-exist. The black point represents the standard values for *σm* and *σw* and the black line shows parameter combinations where *σm/σw* = *sm/sw* = 1.5.

Fig. 6 shows a diagram similar to Fig. 5, but studying the influence of the rescaled growth parameters *σm* and *σw* on system stability. As one would intuitively expect a high growth rate of wild-type relative to mutants (*σw/σm*, which is equal to *sw/sm*), confers an overall advantage to wild-type (upper left area). If this ratio declines the system first reaches an area of stable co-existence and then a region of parameter space that leads to unlimited accumulation of mutants and collapse of the system. The plot also features a black line, indicating where the ratio of growth rates (*σm/σw* = *sm/sw*) is constant (equal to 1.5). Interestingly, this line crosses the area of stable co-existence, indicating that not only the relative growth advantage of mutants decides about their fate, but also the absolute values. High synthesis rates stabilize the system, while low rates de-stabilize it. Fig. 6 has been calculated using the standard parameter values of Table 1. Variations to those parameter values do of course influence the size of the region of stable co-existence. Lower fusion rates (kfus), larger fission rates (kfis), lower fusion rates for mutants (sf), and a lower specificity of degradation (sd) would increase the area of stable co-existence compared to our standard values.

**Fig. 7.**
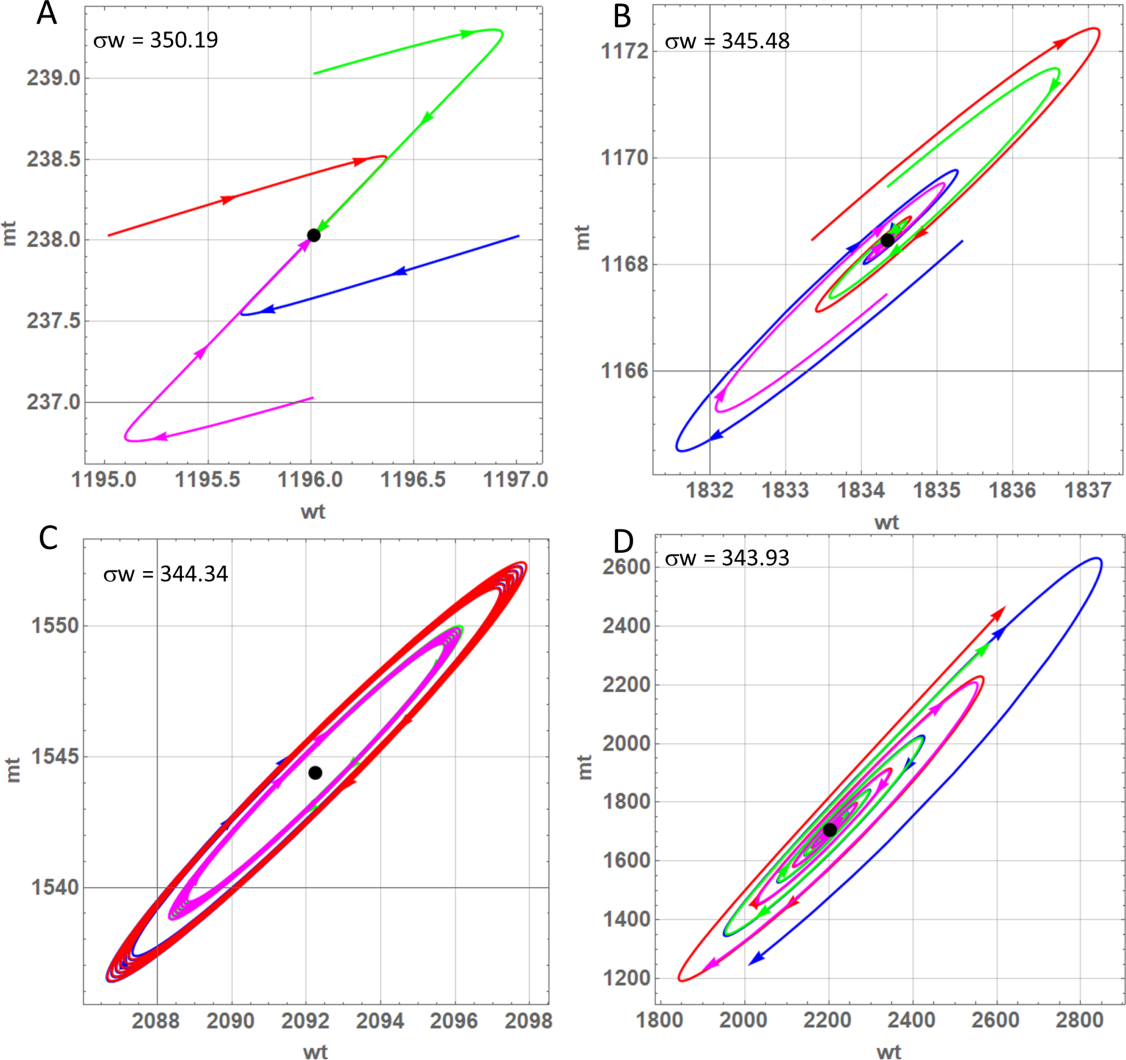
Trajectories of wild-type and mutant mtDNAs for different values of the rescaled growth rate of wild-type, *σw*. The trajectories start one mtDNA “unit” away from the fixpoint and are then attracted or repelled from the fixpoint, which turns into a stable node (A), stable focus (B), unstable focus surrounded by a stable limit cycle (C), or unstable focus with diverging trajectories (D) depending on the value of the parameter.

To better understand the system behavior, it is instructive to study the trajectories of mutant and wild-type mtDNAs close to a fixed point as it changes its character (from stable to unstable), which we enforce by lowering the value of *σw*. Fig. 7A shows that for a value just above the standard value of *σw* (as indicated by the black dot in Fig. 6), the fixed point is a stable node attracting trajectories from all directions. As the value is lowered, the point first turns into a stable focus (Fig. 7B) and finally undergoes a supercritical Hopf bifurcation, rendering the fixed point unstable and creating a stable limit cycle (Fig. 7C), the latter corresponding to stable oscillations in a time-course plot. If the rescaled growth rate is lowered even further, the stable limit cycle grows and gets eventually destroyed in a homoclinic bifurcation, rendering the whole system unstable (Fig. 7D). This leads to a collapse of the system (i.e. unlimited growth of mutant and death of the cell) and marks the lower boundary of the area of stable coexistence for the parameters considered here. The lower bound of this homoclinic bifurcation is shown in Fig. 6 and indicates where the co-existence fixed-point solution diverges (see the Supplementary Material).

To get a better feeling for the biological processes that lead to the behavior of mutant and wild-type close at the fixed point, we will now discuss the green trajectory in Fig. 7B that starts with one mutant mtDNA above the fixed point. Although the trajectories were calculated with the reduced version of the model, we will describe the events in terms of the equations of the full model since they are easier to interpret biologically.

- The higher number of mutants leads to a decreased fraction of mutant as well as wild-type mtDNAs in the singleton state (Eqs. 10 and 11), which means that the degradation rate is reduced and the half-life increased. The higher mutant number also means a higher ATP consumption and thus a lower ATP level. This translates to increased synthesis terms for both mutant and wild-type mtDNAs. Consequently the green trajectory starts towards the upper right.
- When more and more wild-type molecules are accumulating the level of ATP is rising, which reduces the synthesis terms until degradation dominates and the numbers both wild-type and mutant mtDNAs are starting to decline.
- Once the numbers of mtDNAs decline, the fraction of mtDNAs in the singleton state surges, which further increase degradation rates.
- The system overshoots now to the lower left of the fixed point until wild-type numbers have fallen so much that the resulting low ATP level again increases the growth terms to a degree that they compensate for degradation. The trajectory reverses and moves again to the upper right.
- The above sequence of events leads to damped oscillations that finally settle into the fixed point. However, this obviously depends critically on the numerical values of the system parameters. As shown in Fig. 7D, the oscillations can also continue to grow until the system collapses if *σw* (and thus *sw*) is too small.

## Discussion

Mutations of the mitochondrial DNA are at the heart of mitochondrial diseases and form the basis for the mitochondrial theory of aging. It is thus of great importance to better understand the life cycle of mitochondria and mtDNA from synthesis to degradation. For this purpose, we developed a mathematical model describing the population dynamics of wild-type and mutant mtDNA molecules inside postmitotic cells. The model combines our ideas on how a transcriptional feedback loop can lead to a replication advantage of mtDNA mutants (22, 28) with reactions describing the fission and fusion process that underlie mitochondrial dynamics (25–27, 32).

This new level of realism allows the mitochondrial life cycle to be studied in much greater detail, which is also reflected by the complex reaction scheme shown in Fig. 1. An important result of the model analysis is the realization that under certain parameter combinations a stable co-existence of wild-type and mutant mtDNA molecules is possible. Under those conditions, the selection advantages of both molecular species are equal and opposite. Important parameters for combatting mutant variants that are (partly) under the control of the cell include the specificity of degradation (*sd*) and specificity of fusion (*sf*). Interestingly, a low fusion rate of mutants not only strongly favors the wild-type, but at the same time also creates and widens the area of stable co-existence (Fig. 5). If mitochondria with mutant mtDNAs fuse as efficiently as those with wild-type molecules (*sf* = 1), then no co-existence is possible, but it would also require a very high specificity of degradation (*sd* > 1.5) to maintain a population of wild-type mitochondria. Thus, it seems reasonable to assume that the cell operates at intermediate values of *sf* and *sd*, which automatically creates a certain area of stable co-existence. We also note that in the model presented above, convergence to stable co-existence, where it occurs, results in a single fixed point or attractor at a particular level of heteroplasmy. In a detailed empirical study of heteroplasmy of the MELAS mutation in samples from affected human subjects, there was a large variation in heteroplasmy levels between individuals and to some extent with age (33). This result should be expected since the model parameters can differ somewhat between individuals and with the state of their cells (e.g. cellular energy demand), with consequent changes to the predicted fixed point (results not shown).

Another view on the system is provided by Fig. 6 that investigates the effects of different mtDNA replication parameters (*σm, σw*, respectively, *sm, sw*) with *sd* and *sf* being fixed. Not surprisingly, a high synthesis rate for wild-type mtDNA compared to mutant mtDNA leads to a system that contains only wild-type, while a low ratio leads to the unbounded accumulation of mutants. Between these extremes there again exists a zone of stable co-existence, the size of which is influenced by the numerical values of several model parameters such as *kfis, kfus, sd*, or *sf*. Somewhat surprisingly Fig. 6 also shows that not only the ratio of growth parameters is important, but also the absolute values, with low values favoring mutant mtDNAs (while keeping the parameter ratio constant). It turns out that in the area of stable co-existence lower absolute values of the growth parameters can maintain a higher steady-state value of all model variables (including mutants), because the higher steady state means a lower fraction of singletons, which means a lower degradation rate. Conversely this means that mutants, which exist in stable co-existence (as in mitochondrial diseases) might be eradicated if the replication rates of all mtDNAs (irrespective of whether they are wild-type or mutant) could somehow be be increased. When studying how the stable fixpoint becomes unstable, we observed that in principle also stable oscillations are possible (Fig. 7C). This phenomenon, however, is most likely of no biological importance since it exists only for very limited parameter values and the resulting length of the oscillations is in the order of almost 10 y (data not shown).

Although there have been some models that combined fission and fusion with replication and degradation (29–32), none of these observed a stable co-existence of wild-type and mutant mtDNAs. The reason is probably that in these models mutants did not have a replication advantage and that the number of mtDNAs or their synthesis rate was fixed. Including ATP as an important variable that eventually controls the level of mitochondria is not only more realistic, but might also allow for the necessary flexibility in the amounts of mutant and wild-type mtDNAs to display the phenomenon of stable co-existence. Furthermore, although we utilize the same reactions for fusion and fission as Aryaman et al. (32), their emphasis was specifically on the analysis of heteroplasmy variance under neutral selection.

Technically, it could be argued that random drift is also able to produce a long-term co-existence of mutant and wild-type genomes, but as has been shown by simulation studies, random drift cannot explain the accumulation of mitochondrial mutants in short-lived species with the observed low degree of heteroplasmy (21, 22). The concept of random drift requires that replication, degradation as well as fission and fusion of mitochondria are selectively neutral, but current understanding of mitochondrial quality control mechanisms clearly shows that cells actively select against defective organelles via differential fusion and preferential degradation (25–27). Nevertheless, in cases such as mitochondrial tRNA mutations, the situation might be different since tRNA molecules can freely diffuse within the mitochondrial network, which makes it difficult for the fission and fusion based mitophagy system to recognise and remove them (34). These issues merit further study, but our focus in the present work has been to demonstrate how selection alone can explain stable co-existence of mutant and wild-type genomes.

A puzzling observation is that mtDNA mutants are involved in mitochondrial diseases as well as in aging, despite the apparent differences of these processes. Healthy individuals are born with cells containing almost exclusively wild-type mitochondria. Over the course of lifetime, new somatic mtDNA mutations occur in some cells resulting in the clonal accumulation of some of these mutant variants. This leads to a mosaic pattern of affected cells, each harbouring a different type of mutant. Although the fraction of affected cells is initially very small, it grows continuously with advancing age, which is thought to contribute to the ever-increasing decline of function and risk of death, which characterizes the aging process. In case of inherited mitochondrial diseases, however, individuals start into life with all cells carrying some fraction of the same mitochondrial mutant. If mitochondrial mutations cause aging even if present only in a relatively small fraction of cells, how is it possible that mitochondrial diseases, in which all cells are affected, are neither rapidly lethal within the lifetime of an individual (accelerated aging) nor prevented from being stably transmitted from generation to generation?

We believe that the possibility of stable co-existence, as revealed by our model, can provide a resolution of this puzzle. As we have seen, some combinations of parameter values permit a stable co-existence of mutant and wild-type mtDNA. However, the model also reveals that the requirements for stable co-existence to occur are quite stringent, as revealed by the fact that the stable zone in parameter space is rather small (although the exact size also depends on the specific parameter values used). This suggests that mutations capable of causing mitochondrial diseases, in which the mutation can persist through the lifetime and across generations, will be rare within the vast range of mtDNA mutations that can occur. However, this fact might not be noticeable because of observational bias. Mutations located in the upper left part of Fig. 6 will quickly be eradicated by the cellular quality assurance system, while mutations in the lower right area accumulate without limit and kill the developing embryo. Thus, there is a selection process that only lets mutations be inherited that allow for a stable co-existence of mutant and wild-type mitochondria. These mutations then manifest themselves as mitochondrial diseases.

## Supplementary Material

pgac192_Supplemental_File

## Data Availability

All data are included in the manuscript and/or Supplementary Material.
