# Supplementary material for: An explanation of how mutant and wild-type mitochondria might stably co-exist in inherited mitochondrial diseases
Source: PNAS Nexus. 2022 Sep 16;1(4):pgac192. doi: 10.1093/pnasnexus/pgac192 (PMC9802247; doi:10.1093/pnasnexus/pgac192)
Supplement: pgac192_Supplemental_File [file pgac192_supplemental_file.docx]

# Supplementary Material

## Steady state solutions

The steady state solutions of the full model with five equations are quite long and convoluted. However, for completeness, we list here the expressions for a situation where only wild-type and ATP is present:

ATP = -(c*(f - v2)*(halfLwts*kfis*sw + (-kfis + sw)*Log(2)))/(c*halfLwts*kfus*sw*v1 + kfis*(-f + v2)*Log(2)))

wtf = c*v1*(halfLwts*kfis*sw + (-kfis + sw)*Log(2))*(c*halfLwts*kfus*v1*(halfLwts*sw - Log(2)) + (f - v2)*Power(Log(2),2))) / (Log(2)*(-(c*halfLwts*kfus*sw*v1) + kfis*(f - v2)*Log(2))*(-(c*halfLwts*kfus*v1) + f*(halfLwts*kfis + Log(2)) - v2*(halfLwts*kfis + Log(2))))

wts = c*halfLwts*v1*(halfLwts*kfis*sw + (-kfis + sw)*Log(2)))/ (Log(2)*(-(c*halfLwts*kfus*v1) + f*(halfLwts*kfis + Log(2)) - v2*(halfLwts*kfis + Log(2))))

and a situation where all types of mtDNA are present:

ATP = -((c*(-(halfLwts*kfis*(-1 + sf)*sm*sw*v2) + f*sf*((-1 + sd)*sm*sw + kfis*(sm - sd*sw))*Log(2) + sd*(-1 + sf)*(kfis - sm)*sw*v2*Log(2)))/(c*halfLwts*kfus*(-1 + sf)*sf*sm*sw*v1 + kfis*(f*sf*(sm - sd*sw) + sd*(-1 + sf)*sw*v2)*Log(2))))

wtf = ((c*halfLwts*kfus*sf*(-(kfis*sm) + kfis*sd*sw + sm*sw - sd*sm*sw)*v1 + kfis*v2*(halfLwts*kfis*(-sm + sd*sw) + sd*(-sm + sw)*Log(2)))*(-(c*halfLwts*kfus*(-1 + sf)*sf*sm*v1*(halfLwts*sw - Log(2))) + Log(2)*(f*sf*(halfLwts*kfis*(-sm + sd*sw) - (-1 + sd)*sm*Log(2)) + (-1 + sf)*v2*(halfLwts*kfis*(sm - sd*sw) + sd*sm*Log(2)))))/(halfLwts*kfus*sm*(c*halfLwts*kfus*(-1 + sf)*sf*v1 - halfLwts*kfis*v2 + halfLwts*kfis*sf*v2 + f*sf*Log(2) - f*sd*sf*Log(2) - sd*v2*Log(2) + sd*sf*v2*Log(2))*(c*halfLwts*kfus*(-1 + sf)*sf*sm*sw*v1 + kfis*(f*sf*(sm - sd*sw) + sd*(-1 + sf)*sw*v2)*Log(2))))

wts = (c*halfLwts*kfus*sf*(-(kfis*sm) + kfis*sd*sw + sm*sw - sd*sm*sw)*v1 + kfis*v2*(halfLwts*kfis*(-sm + sd*sw) + sd*(-sm + sw)*Log(2)))/(kfus*sm*(c*halfLwts*kfus*(-1 + sf)*sf*v1 - halfLwts*kfis*v2 + halfLwts*kfis*sf*v2 + f*sf*Log(2) - f*sd*sf*Log(2) - sd*v2*Log(2) + sd*sf*v2*Log(2))))

mtf = ((kfis*(f - v2)*Log(2)*(halfLwts*kfis*(sm - sd*sw) + sd*(sm - sw)*Log(2)) + c*halfLwts*kfus*v1*(halfLwts*kfis*(-1 + sf)*sm*sw + ((-sd + sf)*sm*sw + kfis*(-(sf*sm) + sd*sw))*Log(2)))*(c*halfLwts*kfus*(-1 + sf)*sf*sw*v1*(halfLwts*sm - sd*Log(2)) + Log(2)*(-(sd**2*(-1 + sf)*sw*v2*Log(2)) + f*sf*(halfLwts*kfis*(sm - sd*sw) + (-1 + sd)*sd*sw*Log(2)))))/(halfLwts*kfus*sd*sw*Log(2)*(c*halfLwts*kfus*(-1 + sf)*sf*v1 - halfLwts*kfis*v2 + halfLwts*kfis*sf*v2 + f*sf*Log(2) - f*sd*sf*Log(2) - sd*v2*Log(2) + sd*sf*v2*Log(2))* (c*halfLwts*kfus*(-1 + sf)*sf*sm*sw*v1 + kfis*(f*sf*(sm - sd*sw) + sd*(-1 + sf)*sw*v2)*Log(2))))

mts = (-(kfis*(f - v2)*Log(2)*(halfLwts*kfis*(sm - sd*sw) + sd*(sm - sw)*Log(2))) + c*halfLwts*kfus*v1*(-(halfLwts*kfis*(-1 + sf)*sm*sw) + (kfis*sf*sm - kfis*sd*sw + sd*sm*sw - sf*sm*sw)*Log(2)))/(kfus*sd*sw*Log(2)*(c*halfLwts*kfus*(-1 + sf)*sf*v1 - halfLwts*kfis*v2 + halfLwts*kfis*sf*v2 + f*sf*Log(2) - f*sd*sf*Log(2) - sd*v2*Log(2) + sd*sf*v2*Log(2))))

## Derivation of the 2 equation system

As described in more detail in the main text, we approximate the dynamics of the five equation model by reducing the dynamics of ATP and the fraction of the respective single and fused-state mtDNA species.

Using the equation for the dynamics of ATP, this leads to the following condition

$$0=f\cdot(wtf+wts)-v_{1}\cdot ATP-v_{2}\cdot(wtf+wts+mtf+mts),$$

which, with the abbreviations w=wtf+wts and m=mtf+mts, yields

$$ATP=\frac{1}{v_{1}}\cdot(f\cdot w-v_{2}\cdot(w+m)).$$

Analogously, we can approximate the fraction of fused and single mtDNA species. Using the expressions Eq. 10 and Eq. 11, we arrive at the dynamics for the total amounts of wild-type and mutant mtDNA species,

$$\frac{dw}{dt}=\frac{sw\cdot w}{1+\frac{ATP}{c}}-\frac{ln2\cdot wts}{halfLwts}=\frac{sw\cdot w}{1+\frac{1}{cv_{1}}\cdot(f\cdot w-v_{2}\cdot(w+m))}-\frac{ln2\cdot kfis\cdot w}{halfLwts\cdot(kfis+kfus\cdot(sf\cdot m+w))},$$

$$\frac{dm}{dt}=\frac{sm\cdot w}{1+\frac{ATP}{c}}-\frac{sd\cdot ln2\cdot mts}{halfLwts}=\frac{sm\cdot m}{1+\frac{1}{cv_{1}}\cdot(f\cdot w-v_{2}\cdot(w+m))}-\frac{sd\cdot ln2\cdot kfis\cdot m}{halfLwts\cdot(kfis+kfus\cdot sf\cdot(m+w))}.$$

Introducing the dimensionless variables f_w_=(f-v)/(v_1_ c), f_m_= -v_2_/(v_1_ c), κ=kfus/kfis, and the two rescaled synthesis rates, σ_w_ = sw halfLwts/ln2 and σ_m_ = sm halfLwts/ln2, we arrive at a rescaled version of the dynamics,

$$\frac{dw}{dt}=\frac{\sigma_{w}\cdot w}{1+f_{w}w+f_{m}m}-\frac{w}{1+\kappa(s_{f} m+w)},$$

$$\frac{dm}{dt}=\frac{\sigma_{m}\cdot m}{1+f_{w}w+f_{m}m}-\frac{s_{d}m}{1+\kappa s_{f}(m+w)},$$

where the time t has been scaled accordingly.

The steady states of the reduced system can now be obtained by setting dw/dt=0 and dm/dt=0. This yields four possible fixed point states:

1. The trivial solution w=0, m=0.
2. The solution w=0, m≠0, with

$$m=\frac{s_{d}-\sigma_{m}}{\kappa+s_{f}\sigma_{m}-s_{d}f_{m}}.$$

3. The solution w≠0, m=0, with

$$w=\frac{1-\sigma_{w}}{\sigma_{w}\kappa-f_{w}}.$$

4. Finally, there exists the co-existence state with

$$w=\frac{1}{\kappa}\frac{(f_{m}-\kappa s_{f})(\sigma_{m}-s_{d}\sigma_{m})}{f_{m}s_{d}\sigma_{w}-f_{m}s_{f}\sigma_{m}-f_{w}s_{d}s_{f}\sigma_{w}+f_{w}s_{f}\sigma_{m}+\kappa s_{f}^{2}\sigma_{m}\sigma_{w}-\kappa s_{f}\sigma_{m}\sigma_{w}},$$

$$m=\frac{1}{\kappa}\frac{f_{w}s_{d}\sigma_{w}-f_{w}\sigma_{m}-\kappa s_{d}\sigma_{w}-\kappa s_{f}\sigma_{m}\sigma_{w}+\kappa s_{f}\sigma_{m}+\kappa s\sigma_{m}\sigma_{w}}{f_{m}s_{d}\sigma_{w}-f_{m}s_{f}\sigma_{m}-f_{w}s_{d}s_{f}\sigma_{w}+f_{w}s_{f}\sigma_{m}+\kappa s_{f}^{2}\sigma_{m}\sigma_{w}-\kappa s_{f}\sigma_{m}\sigma_{w}},$$

The stability of each of such states can be investigated by evaluating the Jacobian, which is given by

$$J=\frac{1}{{(1+f_{w}w+f_{m}m)}^{2}}\left( \begin{matrix} \sigma_{w}(1+f_{m}m) & -\sigma_{w}f_{m}w \\ -\sigma_{m}f_{w}m & \sigma_{m}(1+f_{w}w) \end{matrix} \right)+\frac{1}{{(1+\kappa(s_{f}m+w))}^{2}}\left( \begin{matrix} -1+\kappa s_{f}m & \kappa s_{f}w \\ \kappa s_{d}s_{f}m & -s_{d}(1+\kappa s_{f}w) \end{matrix} \right).$$

At the trivial solution, the Jacobian evaluates to

$$J=\left( \begin{matrix} \sigma_{w}-1 & 0 \\ 0 & \sigma_{m}-s_{d} \end{matrix} \right).$$

This means the trivial solution is stable for σ_w_<1 (or, in the original parameters, for s_w_<ln2/halfLwts) and σ_m_<s_d_ (or, in the original parameters, for s_m_< s_d_ ln2/halfLwts). Since the Jacobian is diagonal at the trivial solution, its eigenvectores are just the unit vectors. This means for σ_m_ < s_d_ at σ_w_ = 1, the trivial solution becomes unstable and the level of wild type mtDNA grows. This leads to the fixed point solution w ≠0, m = 0 through a transcritical bifurcation (discussed below).

Evaluating the Jacobian at the solution w ≠0, m = 0 leads to the eigenvalues

$$\lambda_{1}=\left( \frac{1}{\sigma_{w}}-1 \right)\frac{{(\sigma_{w}\kappa-f_{w})}^{2}}{{(\kappa-f_{w})}^{2}},$$

$$\lambda_{2}=\frac{\sigma_{m}}{\sigma_{w}}\frac{\sigma_{w}\kappa-f_{w}}{\kappa-f_{w}}-s_{d}\frac{\sigma_{w}\kappa-f_{w}}{\sigma_{w}\kappa-f_{w}+s_{f}\kappa+s_{f}\kappa\sigma_{w}}.$$

The first eigenvalue changes sign at σ_w_=1, leading to a bifurcation. In addition, the w ≠0, m = 0 coincides with the trivial solution at σ_w_=1, indicating that this bifurcation is a transcritical bifurcation between these two solutions.

In addition, the solution w ≠0, m = 0 bifurcates at

$$\sigma_{w}=\sigma_{m}\frac{f_{w}-\kappa s_{f}}{s_{d}(f_{w}-\kappa)+\kappa\sigma_{m}(1-s_{f})},$$

which corresponds to the case when the second eigenvalue changes sign. Importantly, for this condition on σ_w_, the solution w ≠0, m ≠ 0 becomes identical to the w ≠0, m = 0 solution. This indicates a transcritical bifurcation between the w ≠0, m = 0 and w ≠0, m ≠ 0 solutions. This curve marks the upper boundary of the stable co-existence region shown in Fig. 6, and, when rearranged for s_d_, the upper boundary shown in Fig. 5. In addition, the signs of the eigenvalues indicate that above this curve (for larger σ_w,_ or for larger s_d_ values, respectively) the w ≠0, m = 0 solution is stable. We find that below this curve, the w ≠0, m ≠ 0 solution is rendered stable (cf. also the numerical results shown in Fig. 7).

Finally, the fixed point solution w ≠0, m ≠ 0 diverges at

$$\sigma_{w}=\sigma_{m}\frac{s_{f}(f_{m}-f_{w})}{s_{d}(f_{m}-f_{w}s_{f})+\kappa\sigma_{m}(s_{f}^{2}-s_{f})}.$$

Since this curve indicates an unphysical solution, it is shown as the lower boundary of the stable co-existence region as shown in Fig. 6 (and in Fig. 5, but as s_d_ expressed as a function of s_f_).

However, as it can also be observed in the numerical examples shown in Fig. 7, additional bifurcations of the w ≠0, m ≠ 0 occur for some parameters between the two boundary curves discussed above. In particular, for some parameters, the stable w ≠0, m ≠ 0 undergoes a supercritical Hopf bifurcation, creating a stable limit cycle solution. We numerically observe that this limit cycle solution grows in amplitude with smaller σ_w_ values, until it eventually forms a homoclinic loop with the w ≠0, m = 0 solution (not shown here). We are currently working on a detailed analysis of this bifurcation scenario, which is beyond the scope of the present article.
